# Neighborhood Self-Selection: The Role of Pre-Move Health Factors on the Built and Socioeconomic Environment

**DOI:** 10.3390/ijerph121012489

**Published:** 2015-10-08

**Authors:** Peter James, Jaime E. Hart, Mariana C. Arcaya, Diane Feskanich, Francine Laden, S.V. Subramanian

**Affiliations:** 1 Department of Epidemiology, Harvard TH Chan School of Public Health, Boston, MA 02215, USA; E-Mail: francine.laden@channing.harvard.edu; 2 Department of Environmental Health, Harvard TH Chan School of Public Health, Boston, MA 02115, USA; E-Mails: jaime.hart@channing.harvard.edu (J.E.H.); francine.laden@channing.harvard.edu (F.L.); 3 Channing Division of Network Medicine, Department of Medicine, Brigham and Women’s Hospital, Harvard Medical School, Boston, MA 02215, USA; E-Mail: hpdif@channing.harvard.edu; 4 Department of Social and Behavioral Sciences, Harvard TH Chan School of Public Health, Boston, MA 02115, USA; E-Mails: marcaya@hsph.harvard.edu (M.C.A); svsubram@hsph.harvard.edu (S.V.S.)

**Keywords:** residential self-selection, built environment, socioeconomic status, body mass index, physical activity

## Abstract

Residential self-selection bias is a concern in studies of neighborhoods and health. This bias results from health behaviors predicting neighborhood choice. To quantify this bias, we examined associations between pre-move health factors (body mass index, walking, and total physical activity) and post-move neighborhood factors (County Sprawl Index, Census tract socioeconomic status (SES)) in the Nurses’ Health Study (n = 14,159 moves from 1986–2008). Individuals in the highest quartile of pre-move BMI (BMI > 28.4) compared to the lowest quartile (BMI < 22.5) moved to counties that averaged 2.57 points lower on the sprawl index (95% confidence interval −3.55, −1.59) indicating that individuals moved to less dense counties; however, no associations were observed for pre-move walking nor total physical activity. Individuals with higher pre-move BMI tended to move to Census tracts with lower median income and home values and higher levels of poverty. Analyses examining the change in neighborhood environments after a move demonstrated that healthy pre-move behaviors were associated with moves to worse socioeconomic environments. This type of self-selection would bias results downward, underestimating the true relationship between SES and physical activity. Generally, the magnitudes of associations between pre-move health factors and neighborhood measures were small and indicated that residential self-selection was not a major source of bias in analyses in this population.

## 1. Introduction

Stark health differences exist between neighborhoods, with life expectancies differing up to 25 years between zip codes only miles apart [1]. Theories of disease distribution that view contexts as important for health, including ecosocial theory [2], have promoted research on how neighborhood characteristics may contribute to these differences. For example, lower neighborhood-level socioeconomic status (SES) has been consistently linked to negative health outcomes, including physical inactivity and obesity [3,4,5]. Residential segregation by SES serves to distribute resources unevenly between neighborhoods, which can drive neighborhood differences in diet and health behaviors [6]. Substantial evidence demonstrates that physical inactivity and obesity are linked to areas with high levels of urban sprawl, characterized by low residential density and roads with large blocks and poor access [7,8,9,10]. Transportation planning literature indicates that sprawl increases automobile reliance and limits routine physical activity, including walking and bicycling [11]. In short, physical inactivity and obesity have been attributed to features of the neighborhood socioeconomic and built environment; however, the causal nature of these relationships has been questioned. 

Neighborhood and health research to date has been dominated by cross-sectional designs, which are vulnerable to residential self-selection bias resulting from health-related attitudes, neighborhood preferences, or other unmeasured characteristics related to both neighborhood choice and health-related outcomes [12,13,14]. If healthy individuals select neighborhoods based on their preference for health promoting amenities, this self-selection can induce bias that inhibits the establishment of causal relationships between neighborhood factors and health outcomes [15]. Residential self-selection can potentially inflate observed associations, and estimating the magnitude of bias due to selection of persons into neighborhoods is a fundamental methodologic challenge in neighborhood-health research [6,16]. Because experimental studies randomizing individuals to neighborhoods are unethical and unfeasible, accounting for self-selection bias is the most effective approach for observational studies of neighborhoods and health. Concerns over residential self-selection can be alleviated by understanding predictors of mobility into different types of neighborhoods, which can help identify improved strategies to account for these factors in observational studies [6].

Few studies have been able to examine the relationship between mobility patterns and health status, and results are inconsistent [17,18]. A recent analysis by Jokela [19] examined a prospective cohort over 10 years and found that health factors predicted the types of neighborhoods that individuals moved into over followup. Associations between neighborhood disadvantage and health were mostly attributable to between-person differences. The author, as well as a commentary [20], interpreted findings to mean that confounding by individual factors drives any between neighborhood differences in health and called into question research on neighborhoods and health. Conversely, Arcaya *et al*. [21] examined neighborhood outcomes among a group of low-income, involuntarily displaced natural disaster survivors. They found that poor health was predictive of living in a poorer neighborhood years after displacement, and stressed the importance of conceptualizing neighborhood and health relationships as bidirectional over time. Findings across studies may vary due to the country of analysis, as well as the age, race or, socioeconomic status of study participants. This underscores the complexity of neighborhood health research and why more research on self-selection is required in diverse cohorts.

To provide more insight into the magnitude of residential self-selection bias within a longitudinal study, this study aims to examine the relationship between pre-move health factors and subsequent neighborhood features among participants of a long-term prospective cohort study of adult female nurses with a large amount of residential mobility. We aimed to explore whether there was evidence of self-selection in this large prospective cohort study where numerous analyses of neighborhood-health relationships are being investigated. As we progress with neighborhood analyses, this study was important to estimate the potential magnitude of self-selection in this cohort. Additionally, nurses are more likely to have knowledge of health behaviors than the general public. As such, we would expect the potential for self-selection by health to be high in this cohort. We investigated residential self-selection in studies of the built environment and health by analyzing pre-move health factors and the post-move socioeconomic and built environment, measured through, median income, median home value, percent poverty, and the county sprawl index. We hypothesized positive associations would exist between pre-move healthy behaviors and posited “health-promoting” neighborhood features, indicating a potential for confounding by residential self-selection in this cohort.

## 2. Experimental Section

### 2.1. Population

The Nurses’ Health Study (NHS) is an ongoing prospective cohort of 121,700 female nurses who enrolled in 1976 when they were 30–55 years of age. Participants complete mailed biennial questionnaires to provide information on potential risk factors for chronic disease and response rates are over 90% for each follow-up cycle. Ninety-four percent of mailing addresses have been geocoded to the county level. For this analysis, moves were defined as a change of residential addresses to a different county between questionnaire mailings over the period of 1986–2008. The analytical population for this study excluded all women who died prior to follow-up, did not have a pre- and post-move address geocoded at the county level, had missing data on weight and/or physical activity, or did not change county of residence over follow-up. Of the whole cohort, 70.49% did not move counties over followup and were excluded from this analysis. Of those who did move counties at least once over followup (1986–2008), 94.6% moved 3 times or fewer (Supplemental Table 1). Therefore, we examined the first move of each participant as our unit of analysis. When compared to the full cohort, participants who moved counties over follow-up were more likely to be white, had a lower BMI, and higher levels of physical activity (data not shown). Additionally, they lived in denser areas with a higher SES compared to the full cohort. The study was approved by the Institutional Review Board of Brigham and Women’s Hospital, Boston, MA. Informed consent was implied through return of the questionnaires.

### 2.2. Health Factors

Health factors were based on questionnaire responses on self-reported body weight and height, total recreational physical activity (metabolic equivalent hours per week (MET Hrs/Wk)), and walking (MET Hrs/Wk). Pre-move responses were based on the biennial questionnaire prior to a change in address. We calculated BMI (kg/m^2^) from self-reported weight at each questionnaire and self-reported height in the baseline questionnaire. A validation study of 184 NHS participants showed that self-reported weights were highly correlated with measured weights (r = 0.96; mean difference = 1.5 kg) [22], indicating that this self-reported measure is valid in this cohort. 

Each questionnaire, excluding 1990 and 2002, included a question on average time spent per week walking during the past year. Each questionnaire, excluding 1990, 2002, and 2006, included a section on recreational physical activity during the past year. Although the specific activities varied on each questionnaire, questions included the average time per week spent walking, jogging (>10 min per mile), running (≤10 min per mile), bicycling, lap swimming, playing tennis, playing squash or racquet ball, using a rowing machine, and engaging in calisthenics, aerobics, or aerobic dance. Each participant also reported the number of flights of stairs that she climbed daily and her usual walking pace. We multiplied the reported time spent weekly at each activity by its typical energy expenditure requirements expressed in metabolic equivalents (METs), then summed all the activity figures to yield a MET hours per week score [23]. One MET, the energy expended while sitting, is equivalent to 3.5 mL of oxygen uptake per kilogram of body weight per minute for a 70-kg adult. In a validation study of 147 nurses who completed the same physical activity questionnaire concurrent with quarterly 7-day activity diaries, the Pearson correlation coefficient between the MET scores from the questionnaires and the average of the diaries was 0.79 [24]. Walking was calculated based on MET Hrs/wk from the walking question, while total physical activity was calculated based on the sum of all MET Hrs/wk from all activities asked at each questionnaire. 

### 2.3. Neighborhood Socioeconomic Status

Geocoded mailing addresses were linked to 2000 Census tracts [25]. Pre- and post-move Census tract median home value, median income, and percent of population below poverty were recorded as measures of neighborhood SES from the 2000 US Census. 

### 2.4. County Sprawl Index

The county-level sprawl index was developed by Smart Growth America [26] and calculated for all 952 metropolitan counties or statistically equivalent entities in the United States [7]. The index measured two characteristics of sprawl in each county, low residential density and poor street accessibility, derived from six variables in the 2000 US Census. Through principal components analysis, the six Census variables were combined to form one factor that explained 63.4% of the total variance. This factor was then transformed into an index with a mean of 100 and a standard deviation of 25, where higher county sprawl index values indicate a more compact, less sprawling county. We assigned a county sprawl index value to each nurse according to the geocoded county of residence at each questionnaire return. At baseline in 1986, 91.1% of participants lived in counties with a valid county sprawl index value. We excluded from analysis all nurses who lived in counties where the sprawl index was not calculated. 

### 2.5. Statistical Analysis

To examine the magnitude of the relationship between pre-move health factors and subsequent neighborhood environments, we conducted linear regression with each neighborhood measure as the dependent variable separately using a methodology similar to Arcaya *et al*. [21]. Because the number of physical activity questions varied on each questionnaire, leading to uncertain comparability of absolute values across years, we used quartiles of health measures for our analysis. We also ran sensitivity analyses by CDC cutpoints for normal weight (BMI < 25), overweight (BMI 25–30), and obese (BMI > 30). For each model, coefficients and 95% confidence intervals (CI) represented the average predicted values of the neighborhood measures for each quartile of pre-move health factor. Analyses were adjusted for pre-move age and for pre-move neighborhood measures to account for baseline neighborhood factors. Additionally, we re-ran each model using the pre- to post-move change in neighborhood measure as the dependent variable to examine the effect of improving or worsening the neighborhood environment for each individual. For both models, we tested for a linear trend using the median values in each quartile for each pre-move health factor. To examine whether there were temporal trends in residential self-selection by health status, we examined moves prior to the year 2000 versus those from 2000 onwards. Data on retirement was not updated over followup; therefore we included additional analyses stratifying by whether moves took place before or after age 65 to examine whether residential self-selection might differ by retirement status. To understand whether self-selection differed by individual SES, we ran stratified analyses based on the participant’s husband’s highest level of education. Data were analyzed in 2014.

## 3. Results and Discussion

In this sample of the NHS, there were 14,159 participants who moved at least once over follow-up from 1986–2008. Approximately 40% of these participants moved two or more times over follow-up (Supplemental Table S1); however, this analysis examines only the first move for each participant. At least one move occurred from each state in the contiguous United States, as well as the District of Columbia. The women who moved were mostly white (94%) and the mean pre-move age was 62 years (Table 1). On average, participants were slightly overweight prior to a move (mean BMI 25.9) and tended to gain weight after a move. Prior to a move, participants undertook an average of 18.9 MET Hrs/Wk of total physical activity and about 7.5 MET Hrs/Wk of walking. Both total physical activity and walking increased after a move. The mean sprawl index value before a move was 114.5, indicating nurses lived in counties that were denser and more accessible than the national average of 100, and participants tended to move to counties that had lower density and accessibility (mean change in sprawl index of −9.4). Census tract median incomes and median home values were high and percent poverty was low compared to national averages [25]; however, participants tended to move to lower income and lower home value tracts with higher poverty.

**Table 1:** Population characteristics of nurses’ health study participants (n = 14,159 participants).

Variable	Pre-Move	Post-Move
	Mean (SD) or %	Mean (SD) or %
Age (Years)	61.8 (8.6)	63.8 (8.6)
Percent White	94.4	94.4
BMI (kg/m^2^)	25.90 (4.87)	26.10 (4.90)
Total Activity (MET Hrs/Wk)	18.93 (23.10)	20.82 (27.11)
Walking (MET Hrs/Wk)	7.53 (9.53)	7.70 (9.50)
County Sprawl Index	114.50 (26.38)	105.13 (21.17)
Census Tract Median Income ($)	$71.31K ($27.20K)	$64.83K ($23.42K)
Census Tract Median Home Value ($)	$202.09K ($139.79K)	$179.38K ($115.86)
Percent Poverty (%)	6.58% (6.05%)	6.75% (5.66%)

Table 2 shows age-adjusted means for each neighborhood measure by quartile of each pre-move health indicator. We observed a statistically significant relationship between pre-move BMI and post-move county sprawl index (*p* < 0.0001), indicating that individuals with higher BMI tended to move to counties that had lower density and accessibility; however, the absolute difference in sprawl index values between quartiles of BMI was small. Individuals in the highest quartile of pre-move BMI (BMI > 28.4) compared to those with the lowest pre-move BMI (BMI < 22.5) moved to counties that were 2.57 points lower on the sprawl index (95% CI −3.55, −1.59). Individuals with higher levels of pre-move BMI were also more likely to move to Census tracts that had lower median home values and lower incomes, but again the absolute differences in neighborhood factors between quartiles of pre-move BMI were small. Compared to individuals with the lowest pre-move BMIs, individuals with the highest pre-move BMIs moved to Census tracts with a median home value $22K lower (95% CI −$27K, −$17K). Small differences were observed for pre-move levels of both activity measures and Census tract median home values. For instance, the highest quartile of pre-move total physical activity (≥25.2 MET Hrs/Wk) was associated with an $11K (95% CI $6K, $16K) higher Census tract median home value compared to the lowest quartile (< 4.0 MET Hrs/Wk) of pre-move total physical activity. Higher pre-move levels of walking and total physical activity were not associated with levels of sprawl, nor Census tract median income. Additionally, no pre-move health factor was associated with neighborhood levels of poverty.

**Table 2:** Age-adjusted predicted means of neighborhood environment based on pre-move health factors in nurses’ health study participants (n = 14,159 participants) ^a^.

Pre-Move Health Factor Quartiles	County Sprawl Index Predicted Mean (95% CI)	Census Tract Median Home Value Predicted Mean ($) (95% CI)	Census Tract Median Income Predicted Mean ($) (95% CI)	Census Tract Percent Below Poverty Predicted Mean (%) (95% CI)
**BMI**				
Quartile 1 (<22.5)	106.22	$190.04K	$67.25K	6.65%
	(105.53, 106.9)	($186.46K, $193.61K)	($66.51K, $67.99K)	(6.46%, 6.83%)
Quartile 2 (22.5, 24.9)	105.33	$182.83K	$65.47K	6.67%
	(104.63, 106.03)	($179.20K, $186.47K)	($64.71K, $66.22K)	(6.48%, 6.86%)
Quartile 3 (24.9, 28.3)	105.28	$176.68K	$64.01K	6.81%
	(104.6, 105.96)	($173.16K, $180.21K)	($63.27K, $64.74K)	(6.63%, 6.99%)
Quartile 4 (>28.4)	103.65	$167.84K	$62.58K	6.86%
	(102.95, 104.34)	($164.22K, $171.45K)	($61.83K, $63.33K)	(6.67%, 7.05%)
*p* for Trend	**<0.0001**	**<0.0001**	**<0.0001**	0.0746
**Pre-Move Total Physical Activity**				
Quartile 1 (<4.0 MET Hrs/wk)	105.20	$174.93K	$64.29K	6.78%
	(104.50, 105.90)	($171.30K, $178.55K)	($63.53K, $65.04K)	(6.59%, 6.97%)
Quartile 2 (4.0, 11.0 MET Hrs/wk)	104.60	$174.37K	$64.72K	6.59%
	(103.91, 105.28)	($170.81K, $177.93K)	($63.98K, $65.45K)	(6.41%, 6.77%)
Quartile 3 (11.1, 25.1 MET Hrs/wk)	105.24	$182.17K	$65.49K	6.73%
	(104.54, 105.93)	($178.57K, $185.77K)	($64.74K, $66.24K)	(6.54%, 6.91%)
Quartile 4 (>25.2 MET Hrs/wk)	105.48	$186.00K	$64.82K	6.89%
	(104.79, 106.16)	($182.43K, $189.58K)	($64.08K, $65.56K)	(6.71%, 7.07%)
*p* for Trend	0.237	**<0.0001**	0.4132	0.1119
**Pre-Move Walking**				
Quartile 1 (<1.2 MET Hrs/wk)	105.09	$175.84K	$64.60K	6.79%
	(104.38, 105.79)	($172.19K, $179.50K)	($63.84K, $65.36K)	(6.61%, 6.98%)
Quartile 2 (1.7, 3.1 MET Hrs/wk)	104.84	$175.24K	$64.48K	6.68%
	(104.12, 105.55)	($171.53K, $178.95K)	($63.71K, $65.25K)	(6.49%, 6.87%)
Quartile 3 (3.8, 7.5 MET Hrs/wk)	104.97	$179.12K	$64.76K	6.59%
	(104.26, 105.67)	($175.47K, $182.77K)	($64.00K, $65.52K)	(6.40%, 6.78%)
Quartile 4 (>7.5 MET Hrs/wk)	105.54	$186.01K	$65.37K	6.90%
	(104.89, 106.18)	($182.64K, $189.38K)	($64.67K, $66.07K)	(6.72%, 7.07%)
*p* for Trend	0.1831	**<0.0001**	0.0713	0.1375

Note: ^a^ All analyses adjusted for pre-move age in years and pre-move neighborhood environment.

Table 3 shows results of analyses estimating the association between pre-move health factors and change in the neighborhood environment from pre- to post-move. Higher pre-move BMI was associated with a decrease in the sprawl index (indicating moves to less dense, less accessible counties), while there were no associations observed between either pre-move activity measure and the sprawl index. Pre-move health behaviors were not associated with changes in median home value and pre-move BMI was not associated with changes in Census tract median income or percent below poverty. Higher levels of pre-move total physical activity and walking were associated with moves to Census tracts with lower median incomes and higher levels of percent below poverty. For instance, participants in the highest quartile of pre-move walking (> 7.5 MET Hrs/wk) increased their Census tract percent below poverty by 0.42% (95% CI 0.18%, 0.66%).

Sensitivity analyses examining the relationship between pre-move BMI by CDC cutpoints for overweight and obese were similar to those using quartile cutpoints (Supplemental Table S2). Supplemental Table S3 shows results stratified by whether a participant moved prior to the year 2000 or after 2000 for county sprawl index and Census tract median home value. Results were consistent over both time periods. Analyses stratified by whether a participant was above retirement age (65 years) are shown in Supplemental Table S4. We observed no difference in the relationship between pre-move health factors and sprawl index or median home value comparing moves occurring in those under 65 to those who were 65 and older, indicating that retirement did not appear to alter residential self-selection. There were no differences in patterns of residential self-selection by individual-level SES, as seen in analyses stratified by whether a participant’s husband had greater than a high school degree (Supplemental Table S5).

In this analysis of residential mobility among a nationwide study of adult women, we found that higher adiposity individuals tended to move to lower density counties with lower SES and those with lower levels of physical activity tended to move to lower SES neighborhoods. In general, however, the magnitudes of these associations were small, indicating low levels of residential self-selection in this population. Additionally, analyses examining changes in neighborhood built and socioeconomic environment after a move suggested that healthy pre-move behaviors were associated with moves to worse socioeconomic environments. This type of self-selection would bias results downward, underestimating the true relationship between SES and physical activity. Sensitivity analyses demonstrated that results were consistent when examining BMI by CDC cutpoints, as well as when stratifying by the calendar time of a move and by age of a move. As such, this analysis provides evidence that residential self-selection is not a major source of bias in cross-sectional studies of neighborhoods and health for this population.

**Table 3:** Age-adjusted predicted mean change in neighborhood environment based on pre-move health factors in nurses’ health study participants (n = 14,159 participants) ^a^.

Pre-Move Health Factor Quartiles	Change in Sprawl Index Predicted Mean (95% CI)	Change in Census Tract Median Home Value Predicted Mean ($) (95% CI)	Change in Census Tract Median Income Predicted Mean ($) (95% CI)	Change in Census Tract Percent Below Poverty Predicted Mean (%) (95% CI)
**Pre-Move BMI**				
Quartile 1 (<22.5)	−8.57	−$24.42K	−$6.45K	0.27%
	(−9.61, −7.54)	(−$29.29K, −$19.56K)	(−$7.48K, −$5.42K)	(0.02%, 0.53%)
Quartile 2 (22.5, 24.9)	−8.87	−$20.16K	−$6.38K	0.23%
	(−9.93, −7.82)	(−$25.13K, −$15.20K)	(−$7.43K, −$5.33K)	(−0.03%, 0.49%)
Quartile 3 (24.9, 28.3)	−8.87	−$21.16K	−$6.59K	0.19%
	(−9.89, −7.85)	(−$25.98K, −$16.35K)	(−$7.61K, −$5.58K)	(−0.07%, 0.44%)
Quartile 4 (>28.4)	−11.21	−$25.10K	−$6.50K	−0.03%
	(−12.25, −10.16)	(−$30.03K, −$20.17K)	(−$7.54K, −$5.46K)	(−0.28%, 0.23%)
*p* for Trend	**0.0003**	0.643	0.9026	0.0900
**Pre-Move Total Physical Activity**				
Quartile 1 (<4.0 MET Hrs/wk)	−9.38	−$20.53K	−$5.44K	−0.05%
	(−10.43, −8.33)	(−$25.47K, −$15.59K)	(−$6.48K, −$4.40K)	(−0.31%, 0.21%)
Quartile 2 (4.0, 11.0 MET Hrs/wk)	−9.78	−$23.32K	−$6.13K	0.01%
	(−10.81, −8.75)	(−$28.17K, −$18.47K)	(−$7.16K, −$5.11K)	(−0.24%, 0.26%)
Quartile 3 (11.1, 25.1 MET Hrs/wk)	−9.14	−$23.87K	−$6.68K	0.30%
	(−10.18, −8.10)	(−$28.77K, −$18.96K)	(−$7.72K, −$5.65K)	(0.04%, 0.55%)
Quartile 4 (>25.2 MET Hrs/wk)	−9.17	−$23.09K	−$7.66K	0.41%
	(−10.20, −8.14)	(−$27.96K, −$18.23K)	(−$8.68K, −$6.63K)	(0.15%, 0.66%)
*p* for Trend	0.5675	0.6236	**0.0026**	**0.0061**
**Pre-Move Walking**				
Quartile 1 (<1.2 MET Hrs/wk)	−9.11	−$19.62K	−$5.39K	0.09%
	(−10.17, −8.05)	(−$24.60K, −$14.64K)	(−$6.44K, −$4.34K)	(−0.17%, 0.35%)
Quartile 2 (1.7, 3.1 MET Hrs/wk)	−9.60	−$22.02K	−$6.14K	−0.05%
	(−10.67, −8.52)	(−$27.08K, −$16.97K)	(−$7.20K, −$5.07K)	(−0.32%, 0.21%)
Quartile 3 (3.8, 7.5 MET Hrs/wk)	−9.13	−$24.42K	-$6.87K	0.16%
	(−10.18, −8.07)	(−$29.40K, −$19.45K)	(−$7.91K, −$5.82K)	(−0.10%, 0.42%)
Quartile 4 (>7.5 MET Hrs/wk)	−9.60	−$24.46K	−$7.37K	0.42%
	(−10.58, −8.63)	(−$29.04K, −$19.87K)	(−$8.34K, −$6.40K)	(0.18%, 0.66%)
*p* for Trend	0.5907	0.2190	**0.0093**	**0.0145**

Note: ^a^ All analyses adjusted for pre-move age in years.

Our findings are fairly consistent with research on residential self-selection, which indicates that health factors do not drive neighborhood selection. In studies that used methods to address self-selection, the association between the built environment and physical activity oftentimes were in the expected direction and remained statistically significant after accounting for self-selection [27,28], although there were exceptions [19]. Using data from the Health and Retirement Study, Grafova *et al*. [18] examined the association between changes in neighborhood socioeconomic environments and changes in self-assessed health from 1992 to 2000. This analysis examining changes in neighborhoods over time found that neighborhood economic disadvantage decreased more for individuals in poorer health compared to those in better health among both movers and stayers. This indicates that cross-sectional estimates of the association between neighborhood socioeconomic environments and health are likely underestimated, which is consistent with our finding that healthier individuals tended to move to worse socioeconomic environments. A review of 38 studies examining the built environment and physical activity showed that associations remained statistically significant after accounting for self-selection in almost all cases [29]. The same researchers then used the quasi-longitudinal design and demonstrated that changes in the built environment were associated with changes in walking, adjusted for travel attitudes and neighborhood preferences [29]. Another review of cross-sectional studies that adjusted for neighborhood self-selection found that the associations between specific built environmental attributes and physical activity were somewhat mixed, but neighborhood built environment measures were nevertheless consistently associated with higher physical activity levels even after controlling for neighborhood self-selection [28].

Our analysis is parallel to an analysis by Plantinga and Bernell [30], where the authors found that pre-move BMI was associated with sprawl, although our examination adds a few important aspects. First, our analysis was conducted in a nationwide sample that is over three times larger than the Plantinga and Bernell sample, with high quality covariate information. Our analysis also examined health data from participants at the time two years prior to each move while the Plantinga and Bernell analysis examined whether BMI at ages 14–22 years predicted choice of county sprawl at the age of 35–43 years. Finally, our analysis was conducted amongst female nurses who have a broad knowledge of health and are therefore more likely to self-select their residence based on health factors. 

Jokela recently conducted an analysis on a decade of data from a cohort study across Australia [19]. He found that neighborhood disadvantage was associated with poorer health; however, the observed associations were almost completely due to between-person differences. He interpreted his results to mean that confounding by personal factors drives correlations between neighborhoods and health. These findings differed from what we observed in this study likely due to different populations (adult female nurses versus general population) from different countries (United States versus Australia), as well as different data sources (prospective epidemiologic cohort data versus income and labor survey data). Alternatively, Arcaya *et al*. [21] examined data from a group of survivors who were displaced after Hurricane Katrina. The authors found that although health was not associated with neighborhood poverty before the storm, those with pre-storm health problems tended to move to poorer neighborhoods after the storm. This underscores potential reciprocal relationships between health and neighborhoods.

Within the context of the literature on residential self-selection, our results contribute additional information on how health factors may drive neighborhood choice. SES factors and our sprawl metric had poor correlations (R < 0.282), indicating that neither of these independent constructs were related to pre-move health factors. Our cohort of female adult nurses, who were primarily white, did not exhibit strong neighborhood preferences according to either physical activity or BMI patterns. It may be that the socioeconomic, occupational, or demographic makeup of our participants has contributed to their neighborhood choices to a greater degree than health factors. For instance, the relatively affluent socioeconomic status of our participants may lead them to choose neighborhoods based on housing market forces [31]. Unfortunately, the participants included in this study did not provide information on their reasons for residential mobility.

The current study has some additional limitations. First, the physical activity questions changed over the course of follow-up, which may add misclassification to these pre-move health factors; however, the use of quartiles likely minimizes the impact of this misclassification. Self-reported physical activity measures may under-report certain activities, and particularly for routine walking which may be most closely related to the built environment. This study examines only individuals who chose to move outside of the county and does not focus on health factors that may drive individuals to remain in the same neighborhood over time. In addition, this analysis is limited by a county-level measure of sprawl that is limited to metropolitan counties, and therefore the generalizability of results is limited. As stated, we do not have information on the reasons why participants move, which would provide important insights into the mechanism of residential self-selection. The generalizability of these findings is limited based on demographics of this cohort of older adult female nurses who are primarily white; however, these individuals represent those that have sufficient income and opportunity to choose where they move, plus a professional interest in health. Therefore, this population would be most likely to exhibit residential self-selection by health status. Finally, this analysis only explores the effects of health on residential outcomes, and does not model the dynamic association between health and place over time and space. Future research that conceptualizes health and neighborhood outcomes as correlated trajectories and that elucidates the changing bidirectional relationship between health and neighborhood choice over the life course will be important in clarifying the role of the built environment in causally shaping health outcomes. 

This analysis has numerous strengths, foremost its large number of participants who changed addresses over a long period of follow-up. The prospective nature of the cohort allows us to examine health factors prior to changes in residential addresses, as well as changes in pre- and post-move neighborhood environments. Participants in this study moved to various locations across the nation, with moves occurring across every state in the country, contributing to the generalizability of our findings. 

## 4. Conclusions 

In this analysis of the relationship between pre-move health factors and neighborhood built and socioeconomic environments, we observed evidence of a low magnitude of residential self-selection. This finding indicates that residential self-selection bias may be a minor concern in cross-sectional studies of neighborhoods and health. The small associations between health and neighborhood environment choice observed in this relatively affluent cohort with professional health training suggest that groups with more limited residential mobility and lower health awareness would have lower potential for self-selection. Research on neighborhoods and health is important in elucidating the contextual factors that can influence health, and this analysis demonstrates that residential self-selection likely plays a small and inconsistent role in biasing associations between neighborhoods and health. 

## Supplementary Files

Supplementary File 1
