# Supplementary material for: Neighborhood Self-Selection: The Role of Pre-Move Health Factors on the Built and Socioeconomic Environment
Source: Int J Environ Res Public Health. 2015 Oct 8;12(10):12489–504. doi: 10.3390/ijerph121012489 (PMC4626981; doi:10.3390/ijerph121012489)
Supplement: Supplementary File 1 [file ijerph-12-12489-s001.pdf]

Neighborhood Self-Selection: The Role of Pre-Move Health Factors on the Built and Socioeconomic Environment

Table S1. Number of moves per participant among movers in the nurses' health study from 1986–2008 (n = 14,159 participants) [a].

Number of Moves	Frequency	Percent	Cumulative Frequency	Cumulative Percent
1	8081	57.07	8081	57.07
2	3810	26.91	11,891	83.98
3	1504	10.62	13,395	94.6
4	537	3.79	13,932	98.4
5	164	1.16	14,096	99.56
6	47	0.33	14,143	99.89
7	8	0.06	14,151	99.94
8	7	0.05	14,158	99.99
9	1	0.01	14,159	100

Note: [a] All analyses adjusted for pre-move age in years.

Table S2. Age-adjusted predicted means of neighborhood environments based on pre-move BMI categories in nurses' health study participants (n = 14,159 participants) [a].

Pre-Move BMI Categories	County Sprawl Index	Census Tract Median Home Value	Census Tract Median Income	Census Tract Percent Below Poverty
	Predicted Mean	Predicted Mean ($)	Predicted Mean ($)	Predicted Mean (%)
	(95% CI)	(95% CI)	(95% CI)	(95% CI)
Normal (BMI < 25)	105.76 (105.27, 106.25)	$188.88K ($186.20K, $191.56K)	$66.71K ($66.17K, $67.25K)	6.63% (6.50%, 6.77%)
Overweight (BMI 25–30)	104.79 (104.18, 105.41)	$173.36K ($170.00K, $176.71K)	$63.46K ($62.78K, $64.13K)	6.82% (6.66%, 6.99%)
Obese (BMI > 30)	103.92 (103.09, 104.76)	$163.09K ($158.55K, $167.64K)	$61.92K ($61.00K, $62.83K)	6.93% (6.71%, 7.16%)

Note: [a] All analyses adjusted for pre-move age in years.

Table S3. Age-adjusted predicted means of neighborhood environment based on pre-move health factors stratified by pre- and post-2000 moves in nurses' health study participants (n = 14,159 participants) [a].

Pre-Move Health Factor Quartiles	County Sprawl Index		Census Tract Median Home Value	
	Pre-2000	Post-2000	Pre-2000	Post-2000
	Predicted Mean (95% CI)	Predicted Mean (95% CI)	Predicted Mean (95% CI)	Predicted Mean ($) (95% CI)
**BMI**				
Quartile 1 (<22.5)	106.78 (106.02, 107.54)	104.15 (102.52, 105.78)	$192.58K ($188.64K, $196.51K)	$182.37K ($173.92K, $190.82K)
Quartile 2 (22.5, 24.9)	105.42 (104.63, 106.20)	105.05 (103.46, 106.63)	$184.30K ($180.24K, $188.36K)	$177.39K ($169.15K, $185.63K)
Quartile 3 (24.9, 28.3)	105.45 (104.68, 106.22)	104.36 (102.91, 105.81)	$175.27K ($171.28K, $179.26K)	$179.37K ($171.86K, $186.88K)
Quartile 4 (>28.4)	103.90 (103.10, 104.70)	102.69 (101.27, 104.11)	$168.39K ($164.24K, $172.54K)	$164.73K ($157.36K, $172.10K)
p for Interaction	0.3546		0.1347	
**Pre-Move Total Physical Activity**				
Quartile 1 (<4.0 MET Hrs/wk)	105.45 (104.66, 106.24)	104.35 (102.85, 105.86)	$175.83K ($171.73K, $179.93K)	$171.96K ($164.15K, $179.77K)
Quartile 2 (4.0, 11.0 MET Hrs/wk)	104.90 (104.13, 105.67)	103.53 (102.00, 105.07)	$174.54K ($170.56K, $178.52K)	$174.79K ($166.83K, $182.76K)
Quartile 3 (11.1, 25.1 MET Hrs/wk)	105.61 (104.83, 106.39)	103.77 (102.25, 105.30)	$184.12K ($180.07K, $188.17K)	$174.38K ($166.47K, $182.29K)
Quartile 4 (>25.2 MET Hrs/wk)	105.76 (104.98, 106.53)	104.30 (102.80, 105.80)	$187.22K ($183.19K, $191.24K)	$180.42K ($172.63K, $188.21K)
p for Interaction	0.9463		0.4478	
**Pre-Move Walking**				
Quartile 1 (<1.2 MET Hrs/wk)	105.11 (104.30, 105.91)	104.91 (103.46, 106.35)	$175.85K ($171.66K, $180.05K)	$174.93K ($167.43K, $182.43K)
Quartile 2 (1.7, 3.1 MET Hrs/wk)	105.26 (104.45, 106.06)	103.40 (101.86, 104.94)	$177.49K ($173.30K, $181.68K)	$167.77K ($159.75K, $175.79K)
Quartile 3 (3.8, 7.5 MET Hrs/wk)	105.43 (104.65, 106.22)	103.17 (101.60, 104.74)	$180.12K ($176.04K, $184.21K)	$175.74K ($167.57K, $183.90K)
Quartile 4 (>7.5 MET Hrs/wk)	105.80 (105.08, 106.52)	104.33 (102.82, 105.83)	$186.63K ($182.89K, $190.36K)	$182.94K ($175.09K, $190.78K)
p for Interaction	0.3596		0.5769	

Note: [a] All analyses adjusted for pre-move age in years.

Table S4. Predicted means of neighborhood environment based on pre-move health factors stratified by age 65 in nurses' health study participants (n = 14,159 participants).

Pre-Move Health Factor Quartiles	County Sprawl Index		Census Tract Median Home Value	
	<Age 65	≥Age 65	<Age 65	≥Age 65
	Predicted Mean (95% CI)	Predicted Mean (95% CI)	Predicted Mean (95% CI)	Predicted Mean ($) (95% CI)
**BMI**				
Quartile 1 (<22.5)	107.02 (106.19, 107.86)	104.82 (103.61, 106.02)	$195.42K ($191.09K, $199.75K)	$180.96K ($174.69K, $187.23K)
Quartile 2 (22.5, 24.9)	105.49 (104.62, 106.36)	105.07 (103.87, 106.26)	$187.08K ($182.58K, $191.58K)	$175.13K ($168.95K, $181.32K)
Quartile 3 (24.9, 28.3)	105.47 (104.61, 106.33)	104.78 (103.67, 105.88)	$177.52K ($173.06K, $181.98K)	$173.95K ($168.22K, $179.69K)
Quartile 4 (>28.4)	103.85 (102.97, 104.73)	103.19 (102.05, 104.34)	$169.90K ($165.34K, $174.46K)	$163.47K ($157.55K, $169.40K)
p for Interaction	0.3091		0.1611	
**Pre-Move Total Physical Activity**				
Quartile 1 (<4.0 MET Hrs/wk)	105.31 (104.44, 106.18)	105.04 (103.87, 106.20)	$177.62K ($173.09K, $182.15K)	$170.30K ($164.25K, $176.35K)
Quartile 2 (4.0, 11.0 MET Hrs/wk)	104.94 (104.09, 105.78)	104.03 (102.86, 105.21)	$177.25K ($172.86K, $181.63K)	$169.44K ($163.34K, $175.54K)
Quartile 3 (11.1, 25.1 MET Hrs/wk)	105.79 (104.93, 106.65)	104.20 (103.04, 105.37)	$186.64K ($182.15K, $191.12K)	$173.84K ($167.80K, $179.90K)
Quartile 4 (>25.2 MET Hrs/wk)	105.99 (105.13, 106.85)	104.50 (103.36, 105.64)	$189.84K ($185.36K, $194.32K)	$178.70K ($172.78K, $184.63K)
p for Interaction	0.567		0.7026	
**Pre-Move Walking**				
Quartile 1 (<1.2 MET Hrs/wk)	105.04 (103.87, 106.20)	104.58 (103.42, 105.74)	$170.30K ($164.25K, $176.35K)	$170.40K ($164.38K, $176.42K)
Quartile 2 (1.7, 3.1 MET Hrs/wk)	104.03 (102.86, 105.21)	104.64 (103.43, 105.85)	$169.44K ($163.34K, $175.54K)	$165.85K ($159.56K, $172.13K)
Quartile 3 (3.8, 7.5 MET Hrs/wk)	104.20 (103.04, 105.37)	104.08 (102.87, 105.28)	$173.84K ($167.80K, $179.90K)	$176.92K ($170.67K, $183.17K)
Quartile 4 (>7.5 MET Hrs/wk)	104.50 (103.36, 105.64)	104.48 (103.39, 105.56)	$178.70K ($172.78K, $184.62K)	$178.34K ($172.71K, $183.97K)
p for Interaction	0.5896		0.2104	

Table S5. Age-aAdjusted predicted means of neighborhood environment based on pre-move health factors stratified by husband's highest education in nurses' health study participants (n = 14,159 participants) [a].

Pre-Move Health Factor Quartiles	County Sprawl Index		Census Tract Median Home Value	
	High School or Less	More than High School	High School or Less	More than High School
	Predicted Mean (95% CI)	Predicted Mean (95% CI)	Predicted Mean (95% CI)	Predicted Mean ($) (95% CI)
**BMI**				
Quartile 1 (<22.5)	104.06 (102.62, 105.50)	106.68 (105.74, 107.62)	$172.67K ($165.21K, $180.13K)	$199.95K ($195.05K, $204.85K)
Quartile 2 (22.5, 24.9)	103.71 (102.37, 105.05)	105.40 (104.40, 106.41)	$165.89K ($158.94K, $172.83K)	$191.65K ($186.43K, $196.87K)
Quartile 3 (24.9, 28.3)	103.73 (102.49, 104.98)	105.48 (104.47, 106.49)	$164.45K ($157.99K, $170.90K)	$183.07K ($177.85K, $188.29K)
Quartile 4 (>28.4)	102.21 (101.00, 103.41)	103.71 (102.60, 104.81)	$157.96K ($151.70K, $164.21K)	$176.11K ($170.37K, $181.85K)
p for Interaction	0.9691		0.5678	
**Pre-Move Total Physical Activity**				
Quartile 1 (<4.0 MET Hrs/wk)	103.54 (102.33, 104.75)	105.84 (104.75, 106.92)	$163.98K ($157.68K, $170.29K)	$184.59K ($178.96K, $190.23K)
Quartile 2 (4.0, 11.0 MET Hrs/wk)	102.50 (101.24, 103.77)	104.73 (103.71, 105.75)	$160.87K ($154.31K, $167.43K)	$182.41K ($177.12K, $187.70K)
Quartile 3 (11.1, 25.1 MET Hrs/wk)	103.86 (102.54, 105.18)	105.37 (104.37, 106.37)	$168.87K ($162.02K, $175.71K)	$190.32K ($185.14K, $195.50K)
Quartile 4 (>25.2 MET Hrs/wk)	103.58 (102.15, 105.01)	105.81 (104.86, 106.77)	$165.37K ($157.95K, $172.79K)	$195.57K ($190.61K, $200.54K)
p for Interaction	0.7837		0.6798	
**Pre-Move Walking**				
Quartile 1 (<1.2 MET Hrs/wk)	103.58 (102.32, 104.85)	105.73 (104.65, 106.81)	$164.55K ($157.97K, $171.13K)	$186.14K ($180.53K, $191.75K)
Quartile 2 (1.7, 3.1 MET Hrs/wk)	102.59 (101.29, 103.90)	105.54 (104.46, 106.61)	$162.95K ($156.16K, $169.74K)	$184.35K ($178.79K, $189.92K)
Quartile 3 (3.8, 7.5 MET Hrs/wk)	103.71 (102.37, 105.06)	104.67 (103.65, 105.68)	$166.25K ($159.29K, $173.21K)	$188.35K ($183.08K, $193.62K)
Quartile 4 (>7.5 MET Hrs/wk)	103.51 (102.23, 104.80)	105.77 (104.87, 106.68)	$164.85K ($158.19K, $171.51K)	$193.58K ($188.87K, $198.30K)
p for Interaction	0.4490		0.0779	

Note: [a] All analyses adjusted for pre-move age in years.
